# Whole-Brain Atrophy Differences between Progressive Supranuclear Palsy and Idiopathic Parkinson’s Disease

**DOI:** 10.3389/fnagi.2016.00218

**Published:** 2016-09-13

**Authors:** Carlos Guevara, Katherina Bulatova, Gareth J. Barker, Guido Gonzalez, Nicolas A. Crossley, Matthew J. Kempton

**Affiliations:** ^1^Facultad de Medicina, Universidad de ChileSantiago, Chile; ^2^Department of Neuroimaging, Institute of Psychiatry, King’s College LondonLondon, UK

**Keywords:** progressive supranuclear palsy, idiopathic Parkinson’s disease, whole brain atrophy state, SIENA, SIENAX

## Abstract

**Background**: The absence of markers for ante-mortem diagnosis of progressive supranuclear palsy (PSP), results in this disorder being commonly mistaken for other conditions, such as idiopathic Parkinson’s disease (IPD). Such mistakes occur particularly in the initial stages, when “plus syndrome” has not yet clinically emerged.

**Objective**: To investigate the global brain volume and tissue loss in patients with PSP relative to patients with IPD and healthy controls and correlations between clinical parameters and magnetic resonance imaging (MRI)-derived brain volume estimates.

**Methods**: T1-weighted images were obtained from three groups of Chilean Latin American adults: 21 patients with IPD, 18 patients with PSP and 14 healthy controls. We used Structural Imaging Evaluation with Normalization of Atrophy (SIENAX) to assess white matter, gray matter and whole-brain volumes (normalized to cranial volume). Imaging data were used to analyze putative correlations with the clinical status of PSP and IPD patients using the Unified Parkinson’s Disease Rating Scale Part III (UPDRS III), Hoehn and Yahr (H&Y), the Clinical Global Impression for Disease Severity Scale (CGI-S) and the Frontal Assessment Battery (FAB).

**Results**: PSP patients had significantly lower whole brain volume than both IPD patients and controls. Whole brain volume reduction in PSP patients was primarily attributable to gray matter volume reduction. We found a significant correlation between brain volume reduction and clinical status in the PSP group.

**Conclusions**: At the group level, the whole brain and gray matter volumes differentiated patients with PSP from patients with IPD. There was also significant clinical-imaging correlations with motor disturbances in PSP.

## Introduction

Progressive supranuclear palsy (PSP) is the second most common neurodegenerative movement disorder after idiopathic Parkinson’s disease (IPD). PSP is a debilitating disease characterized by early postural instability, ophthalmoplegia, pseudobulbar palsy, dysarthria, axial rigidity, frontal lobe dysfunction and dementia. PSP exhibits inexorable progression, with a median survival time of between 5 and 10 years (Bower et al., 1997). No disease-modifying treatments have been developed since Steele et al. (1964) described PSP in 1963. The definitive diagnosis of PSP is based on the presence of intracellular deposits of neurofibrillary tangle inclusions composed of abnormally phosphorylated microtubules associated with protein-tau (Verny et al., 1996). It is suspected that abnormal inclusions are causally related to cell death because widespread neuronal loss is generally observed in those areas with tau pathology (Williams et al., 2007).

In contrast, IPD is a synucleinopathy restricted to the substantia nigra and other subcortical brain nuclei, and it is characterized by neural depletion, replacement gliosis and intraneural formation of Lewy bodies. The main clinical manifestations are resting tremor, rigidity, impaired postural reflexes and sustained response to levodopa (L-DOPA) treatment.

Despite these differences, both disorders share some common clinical features, such as akinetic rigidity, making the diagnosis, which is initially based on clinical presentation only, rather difficult, especially in early stages. For the approach to the diagnosis and differential diagnosis of parkinsonian syndromes no quantitative magnetic resonance imaging (qMRI) technique is specifically recommended for routine use in clinical practice (Politis, 2014).

*In vivo* qMRI studies have largely used voxel based morphometry (VBM) to study tissue loss independently of prior (regional) assumptions throughout brains. A recent meta-analysis of gray matter loss in IPD suggests that these patients have reduced volume in the inferior frontal gyrus and precentral gyrus (Shao et al., 2014), but another meta-analysis of gray matter loss reports that IPD does not show any significant distinguished area of atrophy (Yu et al., 2015). By contrary, in PSP, qMRI studies have been more consistent to reflect the ongoing neuronal loss in the lateral orbitofrontal and dorsolateral prefrontal cortices (Brenneis et al., 2004; Cordato et al., 2005; Padovani et al., 2006) and in the thalamus and caudate nucleus (Price et al., 2004; Boxer et al., 2006). Some VBM studies have reported being able to differentiate PSP from IPD at the group level (Price et al., 2004). However, there is no clinical application for VBM. This technique only allows between groups analyses or within group correlations to explore tissue loss independently of prior assumptions throughout the brains.

Here, we are interested in a qMRI technique suitable to be applied at the individual level with a potential clinical utility in the differential diagnosis in parkinsonian syndromes. In this study, we have chosen the Structural Imaging Evaluation with Normalization of Atrophy (SIENAX). SIENAX is an MRI-based algorithm that quantifies loss of brain tissue volume by normalizing the brain volume to the cranial volume (a proxy for premorbid brain volume). The method is fully automated with 0.5–1% brain volume accuracy for single time point (cross-sectional) designs (Smith et al., 2001, 2004). SIENA will be used in patients with IPD and PSP and in healthy controls. Our hypothesis is that IPD at early stages may not have atrophy outside the substantia nigra and that PSP does have widespread tissue loss. Thus global measurement of brain volume and tissue type-specific volumes (i.e., gray and white matter volumes) could be a useful tool to improve the differentiation between IPD and PSP patients.

## Materials and Methods

This project was approved by the local Research Ethics Committees of San Juan de Dios Hospital, Santiago, Chile. Written informed consent was provided by all of the subjects prior to participation in the study.

### Subjects and Clinical Assessments

Three groups of Chilean Latin American adults, including PSP patients (*n* = 18), IPD patients (*n* = 21) and healthy controls (*n* = 14), were selected. The patients were recruited from the Movement Disorders Clinic at Hospital San Juan de Dios, Santiago, Chile. Internationally established operational criteria were used to assess the diagnoses of PSP and IPD (Hughes et al., 1992; Litvan et al., 2003). The clinical characteristics and demographics of each group are shown in Table 1. Every patient was examined by the same clinician (CG) within 1 week of the MRI scan acquisition. None of the patient was treated with cognition enhancing agents. Fourteen IPD patients had the tremor dominant phenotype and seven had the postural instability gait disorder phenotype. All IPD subjects were treated with dopaminergic medication and were examined first on one morning in their “best on” state. Of the 18 PSP patients, 16 had the typical features of classic PSP (Richardson’s syndrome) and two had an atypical profile with tremor and moderate L-DOPA responsiveness (PSP-Parkinsonism variant).

**Table 1:** **Demographics, clinical and volumetric data in idiopathic Parkinson’s disease (IPD), progressive supranuclear palsy (PSP) and control groups**.

	IPD group *n* = 21	PSP group *n* = 18	Control group *n* = 14	Group comparisons	Signifcant pair wise comparisons
Age at examination (years ± SD)	62.6 ± 11.1	67.7 ± 9.0	65.2 ± 6.4	*F* = 1.9; df = 2; *p* = 0.14	
Gender Female (*n*): Male (*n*)	12:9	9:9	9:5	χ^2^ = 0.65; df = 1; *p* = 0.71	
Disease duration (years ± SD)	3.3 ± 3.5	2.8 ± 2.1	–	*p* = 0.26; *t* = 0.54; df = 37; *F* = 5.36
UPDRS III (mean ± SD)	23.0 ± 12.0	44.0 ± 16.0	–	*p* < 0.001; *z* = −3.75
H & Y (mean ± SD)	1.9 ± 0.7	2.8 ± 0.9	–	*p* < 0.001; *z* = −3.25
CGI-S (mean ± SD)	3.5 ± 0.7	4.5 ± 0.7	–	*p* < 0.001; *z* = −3.64
FAB (mean ± SD)	14.5 ± 3.3	10.8 ± 4.8	–	*p* = 0.015; *z* = −2.42
**Normalized brain volumes**					
Whole brain volume (ml ± SD)	1547.0 ± 67.0	1470.0 ± 104.0	1548.0 ± 76.0	*F* = 5.08; df = 2; *p* = 0.01	PSP vs.Controls = 0.031 PSP vs. IPD = 0.016
Gray matter volume (ml ± SD)	775.0 ± 41.0	723.0 ± 85.0	757.0 ± 41.0	*F* = 5.5; df = 2; *p* = 0.007	PSP vs. Controls = 0.01 PSP vs. IPD = 0.031
White matter volume (ml ± SD)	772.0 ± 40.0	731.0 ± 79.0	757.0 ± 40.0	*F* = 2.6; df = 2; *p* = 0.08	

Clinical assessments of both disease groups included the following instruments:

– Hoehn and Yahr Scale (H&Y). This scale was originally designed for the assessment of the general severity of IPD patients. However, it is also widely used for PSP. This simple tool estimates both the transition from unilateral to bilateral motor involvement and the impairment of balance and gait (Hoehn and Yahr, 2001).
– Unified Parkinson’s disease Rating Scale Part III (UPDRS III). This scale is also commonly used in PSP evaluations. Factor analysis has revealed that some of the items of the UPDRS III can be applied in assessing both conditions regarding axial and limb bradykinesia, tremor and rigidity, and face and speech disturbances (Cubo et al., 2000).
– Clinical Global Impression for Disease Severity (CGI-S). This is a short scale that relies upon the clinician’s appraisal of the severity of the disorder. It rates patient’s status on a 1–7 scale, where 1 = “normal, not at all ill”, and 7 = “extremely ill”. It has been suggested that this scale is particularly influenced by motor signs, general disability and cognitive impairment in IPD (Martínez-Martín et al., 2006).
– The Frontal Assessment Battery (FAB). This scale was designed to detect the dysexecutive syndrome at the bedside of patients with extrapyramidal disorders, including PSP (Dubois et al., 2000). It consists of six sub-tests intended to explore individual executive functions: conceptualization, mental flexibility, motor programming, sensitivity to interference of inhibitory control and environmental autonomy. The scale has a maximum of 18 points, with higher scores indicating better performance.

### MRI Acquisition

MRI images were acquired on a 3.0 T Philips Medical System. Axial T1-weighted images, covering the whole brain, were obtained using a 3D inversion recovery prepared spoiled gradient echo (IR-SPGR) sequence. The following parameters were used: repetition time (TR) of 8.1 ms; echo time (TE) of 3.7 ms; inversion time (TI) of 450 ms; voxel size of 0.699 × 0.699 × 1 mm; excitation flip angle of 8^o^; matrix size of 248 × 226; field of view (FOV) of 24 cm; 198 axial 1-mm slices. MRI scans of every patient were assessed by an experienced neuroradiologist (GG) to exclude gross anatomical abnormalities.

### Imaging Processing

All of the data were made anonymous by removing any references to the patient’s name from the image headers and replacing these data with a unique ID. Whole brain volume and brain tissue volumes were estimated using SIENAX (Smith et al., 2001, 2002, 2004). Briefly, SIENAX extracts brain and skull images from the acquired MRI data. The brain image is then affine-registered to Montreal Neurological Institute (MNI) 152 space, using the skull image to determine the registration scaling. The registration scaling is then used to obtain a volumetric scaling factor, which is employed to normalize tissue volume estimates. Segmentation with partial volume estimation is subsequently performed to calculate total volume of brain tissue, including separate estimates of volumes of gray matter and white matter (Smith et al., 2004).

### Statistical Analyses

Analyses of the clinical data and clinical-imaging correlations were performed using the Statistical Package for Social Sciences (SPSS, Inc., Chicago, IL, USA, version 22). The results are presented as the mean ± standard deviation (SD). In all cases, a two-sided *p* value of <0.05 was considered as significant. Visual inspection of the data, using histograms and QQ-plots, was undertaken to check for violations of the assumption of normal distribution. Levene’s test of equal variances was used to verify the assumption of homogeneity of variances. Based on these assessments, parametric or non-parametric statistical tests were then used, as appropriate. Disease duration was compared using a two-tailed *t-*test. Disease severity and cognitive estimations, which were not distributed normally, were assessed using the Mann-Whitney test. The chi-square test for homogeneity was used to compare the distribution of men and women across groups. The associations between MRI-derived measurements and clinical scores were assessed with bivariate correlations. One way analysis of variance (ANOVA) was performed for normally distributed data (age at examination and MRI-derived measures). Tukey’s test was used to control for multiple comparisons.

## Results

### Demographics, Clinical and MRI Variables

The PSP and IPD patients exhibited clinical features typical for their respective diagnoses (Table 1). All 18 PSP cases had progressive symmetric parkinsonism accompanied by postural instability, and 14 had supranuclear ophthalmoplegia. All 21 IPD patients had L-DOPA-responsive akinetic-rigid syndrome. There were no significant differences in age or sex between the groups. The IPD patients had longer disease durations than the PSP patients, while the PSP patients showed greater impairment in the UPDRS III, H&Y, CGI-S and FAB assessments.

### Normalized Brain Tissue Volumes (Table 1, Figure 1)

**Figure 1:**
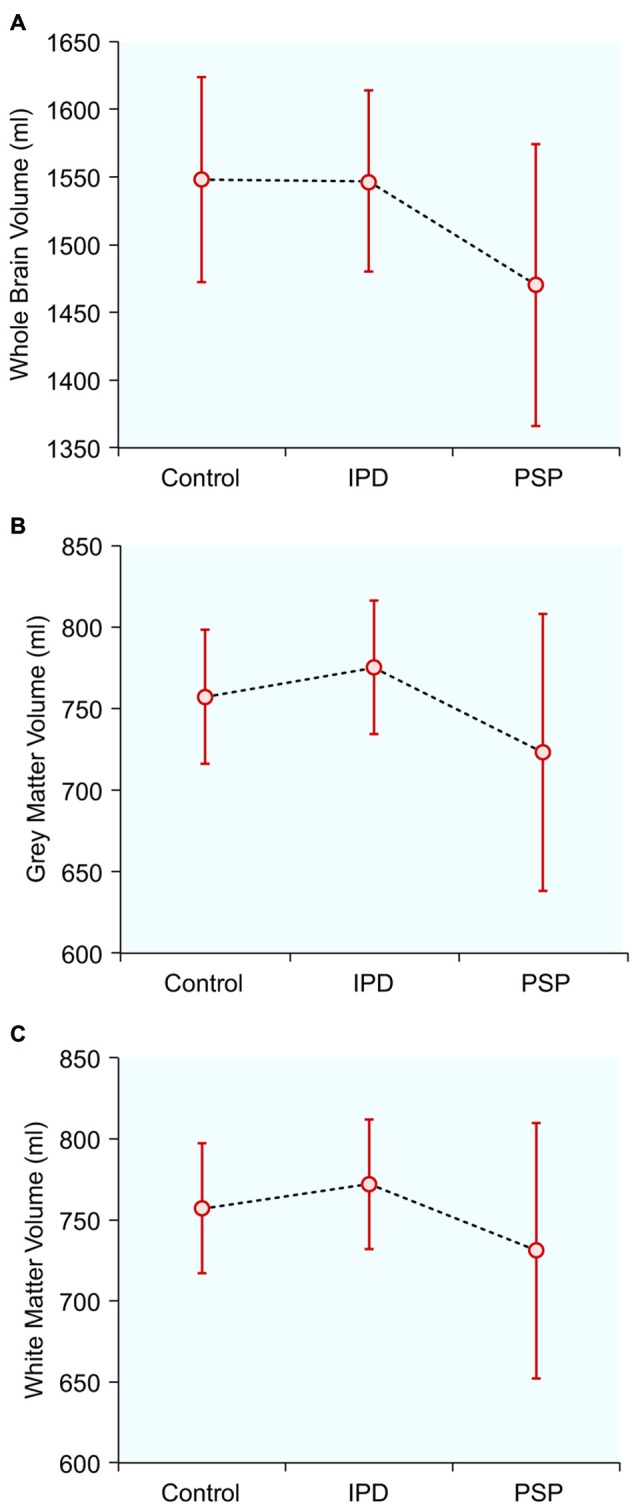
**Normalized brain tissue volumes in controls, idiopathic Parkinson’s disease (IPD) and progressive supranuclear palsy (PSP): (A) Whole brain volume; (B) Gray matter volume; (C) White matter volume**.

The mean whole-brain volume in PSP was significantly lower, compared to the IPD group (*p* = 0.016). The gray matter volume in PSP was also significantly lower than in IPD (*p* = 0.031). The white matter volume did not differ among the study groups. The IPD patients did not differ from the controls in any of these parameters.

### Exploratory Correlations Between Clinical Evaluations and Brain Tissue Volumes (Table 2, Figure 2)

**Figure 2:**
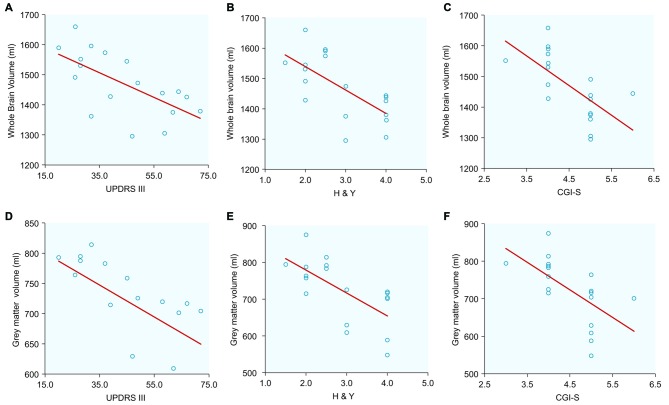
**Correlations between normalized whole brain and gray matter volumes with clinical scores in PSP patients: (A) Whole brain volume and Unified Parkinson’s Disease Rating Scale Part III (UPDRS III); (B) Whole brain volume and Hoehn and Yahr (H&Y); (C) Whole brain volume and Clinical Global Impression for Disease Severity (CGI-S); (D) Gray matter volume and UPDRS III; (E) Gray matter volume and H&Y; (F) Gray matter volume and CGI-S**.

Large (*r* > 0.5) and significant clinical-imaging associations were identified in PSP between the whole-brain volume and the UPDRS III (*r* = −0.645; *p* = 0.004), H&Y (*r* = −0.660; *p* = 0.002) and CGI-S scales (*r* = −0.650; *p* = 0.003). Additionally, the PSP patients exhibited significant correlations between gray matter volume and UPDRS III (*r* = −0.510; *p* = 0.030), H&Y (*r* = −0.660; *p* = 0.003), and CGI-S scores (*r* = −0.610; *p* = 0.007). In PSP, white matter volume was correlated with the UPDRS III (*r* = −0.55; *p* = 0.017) and CGI-S (*r* = −0.65; *p* = 0.003).

No significant correlations were found between whole-brain, gray and white matter volumes with clinical parameters in IPD (Table 2). In both disease groups, neither disease duration nor FAB was correlated with any MRI-derived measurements.

**Table 2:** **Correlations between normalized brain volumes and clinical scores in PSP and IPD**.

Group	PSP		IPD	
	*r*	*p*	*R*	*p*
**Gray matter**
UPDRS III^a^	**−0.510**	**0.030**	−0.39	0.08
H & Y^b^	**−0.660**	**0.003**	−0.30	0.17
CGI-S^c^	**−0.610**	**0.007**	−0.18	0.41
FAB^d^	0.480	0.090	0.27	0.23
Disease duration	−0.340	0.100	0.12	0.57
**White matter**
UPDRS III^a^	**−0.550**	**0.017**	0.14	0.54
H & Y^b^	−0.420	0.080	0.11	0.62
CGI-S^c^	**−0.650**	**0.030**	−0.20	0.91
FAB^d^	0.360	0.200	0.14	0.54
Disease duration	0.060	0.800	0.11	0.60
**Whole brain volume**
UPDRS III^a^	**−0.645**	**0.004**	−0.12	0.60
H & Y^b^	**−0.660**	**0.002**	−0.12	0.60
CGI-S^c^	**−0.650**	**0.003**	−0.13	0.57
FAB^d^	0.480	0.080	0.39	0.08
Disease duration	−0.290	0.240	0.14	0.53

*Bold text indicates significant correlations. ^a^Unified Parkinson’s Disease Rating Scale Part III; ^b^Hoehn & Yahr Scale; ^c^Clinical Global Impression for Disease Severity; ^d^The Frontal Assessment Battery*.

## Discussion

The aim of this study was to explore global brain volume loss in patients with PSP compared to patients with IPD and healthy controls and to evaluate the correlations between clinical parameters and MRI-derived brain volume estimation. Our results showed that patients with PSP had reduced total brain volumes, compared to both IPD patients and controls. Volume loss was mainly due to reductions in gray matter. Furthermore, these changes were significantly related to the clinical findings in PSP. These results suggested that whole-brain quantitative MRI studies could be helpful in differentiating these patients.

Patients with IPD did not show global-brain atrophy. This could be due to a genuine lack of neuronal loss in the early and middle stage, or it could simply reflect that the magnitude of atrophy is not detectable when quantified *in vivo* using this qMRI technique. Furthermore, neither whole brain volume nor gray matter volume showed any association with clinical deterioration in the IPD group in our study. There have been two previous works using SIENAX in IPD patients, and our results are consistent with these studies as no brain volume changes were found when compared with healthy controls (Tessa et al., 2008; Dalaker et al., 2009). Thus, the mean global brain volume in IPD supports the idea that motor deficits in L-DOPA-responsive IPD patients are related predominantly to localized loss of selective dopaminergic neurons in the substantia nigra.

Conversely, the results in the PSP group are according to the pathological data and with previous studies using qMRI techniques. Gray matter and whole-brain volume loss in PSP were correlated with motor disability, as quantified using the UPDRS III, H&Y and CGI-S. Previous studies have reported that UPSRS III score was correlated with atrophy in the caudate and motor cingulate cortices (Cordato et al., 2005). Our qMRI findings were also consistent with studies in PSP patients in which degeneration in both subcortical and cortical sites was correlated with clinical disability (Cordato et al., 2005; Padovani et al., 2006; Tessa et al., 2008; Lagarde et al., 2013).

Patients with PSP scored lower on the FAB test. However, FAB scores were not significantly correlated with gray matter loss. Previous studies have found correlations between FAB scores and localized changes in the frontal lobe, orbitofrontal cortex, midbrain and cerebellum (Cordato et al., 2005; Giordano et al., 2013). Others have found no significant correlations between localized gray matter reductions and cognitive measurements suggesting that reductions are more related to global cortical reductions. Using VBM, Lagarde et al. (2013) recently reported that PSP patients, compared to healthy controls, showed significantly lower gray matter volumes in the left inferior temporal gyrus, right precentral gyrus, right central gyrus, middle temporal gyrus and the anterior nucleus of the right thalamus. However, none of these areas showed a correlation with FAB or with other neuropsychological evaluations, such as the Mattis Dementia Rating Scale (MDRS) and the Mini-Mental State Examination (MMSE). These authors suggested that widespread atrophy of subcortical and cortical gray matter might have prevented them from detecting significant correlations.

In relation with other qMRI techniques, SIENAX estimates global tissue measures and is suitable to be applied at the individual level. This may provide a *potential* utility on the clinical ground, but it is not suitable for detecting localized gray matter atrophy. Regional-specific method (e.g., VBM) have different purposes. VBM has largely been used in an unbiased fashion in these disorders and many sites of neuronal vulnerability have been reported in IPD and PSP. These findings are useful for generating biological hypotheses or suggesting regions of interest for clinical and radiological works.

We think that global tissue-specific measures (e.g., SIENAX) and regional-specific method (f.e VBM) are complementary approaches in neurodegeneration.

A problem with using brain volume as a disease outcome is that it may not reflect physiologic or synaptic health. Furthermore, we do not know if the loss of brain volume might be influenced by causes that are common in people with chronic brain disorders, but only indirectly related to the disease itself, such as minor head trauma, nutritional deficiency or dehydration. Although, these sources of variance are certainly less than those for the clinical measure. Given the actual state of the art in neuroimaging, SIENAX may be among the simplest MRI tools, but complex methodologies do not necessarily lead to robust and coherent results. SIENAX offers several advantages over other quantitative MRI techniques, including high reproducibility of results and the capability to provide a robust measurement of the global changes associated with disease conditions. Moreover, a particular advantage of SIENAX is its relative insensitivity to differences in scanning parameters, making this tool suitable for multicenter studies (Smith et al., 2004).

Although we presented a relatively small number of patients in each group, our findings suggested that SIENAX can be used to quantify *in vivo* brain volume loss in IPD and PSP at the group level. Before the technique can be used diagnostically; however, a greater number of patients and longer prospective follow-up are needed to establish discriminatory cut-off points of these measurements and to estimate sensitivity, specificity and positive predictive values.

## Key Concepts

– SIENAX is an MRI-based algorithm that quantifies brain tissue volume by normalizing the brain volume to the cranial volume. SIENAX extracts the brain and skull images from the acquired MRI data. Segmentation with partial volume estimation is subsequently performed to calculate total volume of brain tissue, including separate estimates of volumes of gray matter and white matter.
– PSP is the second most common neurodegenerative movement disorder after IPD. PSP is a debilitating disease characterized by early postural instability, ophthalmoplegia, pseudobulbar palsy, dysarthria, axial rigidity, frontal lobe dysfunction and dementia. PSP exhibits inexorable progression, with a median survival time of between 5 and 10 years.
– PSP and PD share some common clinical features, such as akinetic rigidity, making the diagnosis rather difficult which is initially based on clinical presentation only, especially in the early stages. Some imaging studies have reported being able to differentiate PSP from IPD, although standard MRI assessment of images is rather insensitive for the estimation of neurodegeneration.
– At the group level, whole brain and gray matter volumes differentiated patients with PSP from patients with IPD.

## Author Contributions

CG, KB, GJB, GG, NAC, MJK have made substantial contribution to the conception or design of the work; or the acquisition, analysis, or interpretation of data for the work; Drafting the work or revising it critically for important intellectual content; Final approval of the version to be published; Agreement to be accountable for all aspects of the work in ensuring that questions related to the accuracy or integrity of any part of the work are appropriately investigated and resolved.

## Conflict of Interest Statement

The authors declare that the research was conducted in the absence of any commercial or financial relationships that could be construed as a potential conflict of interest.
